# Are Some Metals in Tattoo Inks Harmful to Health? An Analytical Approach

**DOI:** 10.1021/acs.chemrestox.2c00323

**Published:** 2022-12-30

**Authors:** Sumru Sozer Karadagli, Islam Cansever, Guliz Armagan, Ozlem Sogut

**Affiliations:** † Department of Pharmaceutical Toxicology, Faculty of Pharmacy, Ege University, Izmir 35040, Turkey; ‡ Central Research Laboratories, Katip Celebi University, Izmir 35620, Turkey; § Department of Biochemistry, Faculty of Pharmacy, Ege University, Izmir 35040, Turkey; ∥ Department of Analytical Chemistry, Faculty of Pharmacy, Ege University, Izmir 35040, Turkey

## Abstract

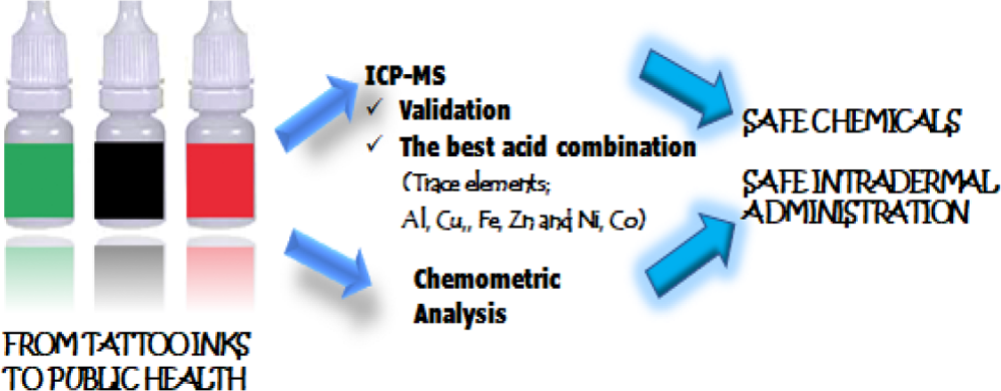

Tattoo application is widely performed all over the world; however, injection of coloring substances into the skin as metals may pose a risk for allergies and other skin inflammations and systemic diseases. In this context, tattoo inks in green, black, and red colors of three brands were purchased. Before starting the analysis, the acid mixture suitable for microwave burning was determined, and according to these results, the inks were digested with nitric acid, hydrochloric acid, and hydrofluoric acid. Then, method validation was performed for tattoo inks using inductively coupled plasma-mass spectrometry. The relative contribution of metals to the tattoo ink composition was highly variable between colors and brands. Elements found in the main components of inks are as follows (in mg kg^–1^): Al, 1191.1–3424.9; Co, 0.04–1.07; Cu, 1.24–2523.4; Fe, 16.98–318.42; Ni, 0.63–17.53; and Zn, 2.6–46.9. It has been determined by the Environmental Protection Agency that in some products, especially the copper element is above the determined limit. The analysis results obtained were classified by chemometric analysis, and the color and brand relationship were determined. More toxicological studies are necessary to understand the effects of tattoo inks containing heavy metals and/or organic components.

## 1 Introduction

Tattoo is an application, which is widely used today, by injecting products consisting of coloring and auxiliary substances into the skin to create a permanent mark on the skin or a visual design.^1^ Tattooing is an important economical market as it is mentioned in the NICNAS.^2^ However, inadequate standardization of ink components is a problem. Today, it is evident that the contents of tattoos are very variable, and they contain both natural and metal salts. Basically, dichromate salts, cobalt (Co), cadmium, and mercury are considered bases for the colors green, blue, yellow, and red, while iron oxide, titanium dioxide, carbon, and manganese are commonly used to create the colors brown, white, black, and violet. Iron oxides are present in 1–4% of all tattoo inks.^3^ In addition, organic pigments and metals (aluminum (Al), calcium, cadmium, etc.) are generally used to obtain different tones and brightness or to lighten the existing colors.^4^

Tattoo inks are not classified as pharmaceutical or cosmetic. The body is directly exposed to the toxic substances contained in the ink due to the injection of tattoo ink into the skin. Pigments may accumulate in the lymph nodes or other organs as they are in direct contact with the skin tissue and lymphatic system.^5^

On the other hand, the analysis of the excess of other elements is also important since they may damage the biological system as well. Accepted levels of elements for tattoo inks have been determined by the Food and Drug Administration (FDA). Accordingly, the recommended limit for soluble copper (Cu) is 25 mg kg^–1^. Limits for other elements apply to their total content and 50 mg kg^–1^ for zinc (Zn) and 25 mg kg^–1^ for Co. CoE ResAP (2008)1 did not define a limit for the presence of nickel (Ni) but emphasized that the concentration should be low enough to be technically detectable. Ni is an allergenic metal. Likewise, limit values of iron (Fe) and Al are not given.^6−9^

Numerous case reports about inflammation associated with pseudolymphoma, allergic or granulomatous skin reactions, and long-term cancer are presented in literature reviews.^7−9^ The CoE ResAP(2008)1 report states that 9% of samples exceeded the recommended maximum concentrations in all ink and cosmetic products analyzed for metal presence. Some of these elements are found in the body essentially, but high doses of Al, Ni, Cu, Co, Fe, and Zn, commonly found in tattoo inks, can produce adverse effects. Therefore, in this study, it is aimed to analyze different trace elements with a validated analytical method and to classify them by chemometry.

## 2 Materials and Methods

### 2.1 Reagents

All the acids used for digestion were Supra-pure grade. Elemental calibration standards were prepared from 10 gmL^–1^ of a multielement stock standard solution (Merck, Darmstadt, Germany). Tri-distilled ultrapure water was used in all steps of the analysis, bi-distilled water was supplied by a Merck Millipore Milli 2 Integral 2 system (Molsheim France), and by using classical distillation apparatus the third distillation was carried out.

### 2.2 Apparatus

An inductively coupled plasma-mass spectrometer (ICP-MS 7800, Agilent, USA) was used for the elemental analysis, and the operating conditions of the ICP-MS are given in Table 1.

**Table 1:** Working Conditions for ICP-MS Detection

ICP-MS parameters	value
plasma mode	general purpose
RF power	1550 W
RF matching	1.80 V
S/C temperature	2 °C
sample depth	10 mm
carrier gas flow rate	1.0 L/min
nebulizer pump flow rate	0.1 rps
internal standards	^6^Li, ^4^5Sc, ^72^Ge, _103_Rh, ^115^In, ^159^Tb, ^175^Lu, ^209^Bi
tuning solvent	^7^Li, ^89^Y, ^205^Tl

### 2.3 Sampling

Three colors Red (r), Green (g), and Black (b) and three brands (A, B, C) of tattoo inks were purchased from Turkish markets, and the color and the brands were chosen according to the popularity of the usage in Turkey. The inks were in liquid form with different viscosity.

Ink samples were microwave-digested (Berghof Speedwave two, Eningen Germany), and a series of studies were conducted to find out the best acid/acid mixtures for degradation of the samples. Each combination was coded as given in Table 2.

**Table 2:** Acid Mixtures Used for the Digestion of Tattoo Inks

I	6 mL HNO_3_ + 3 mL HCl + 0.5 mL HF
II	7 mL HNO_3_ + 1 mL HCl + 1 mL H_2_O_2_
III	8 mL HNO_3_ + 1 mL HF + 1 mL H_2_O_2_
IV	7 mL HNO_3_ + 1 mL H_2_O_2_
V	8 mL HNO_3_ + 1 mL HF
VI	6 mL HNO_3_ + 3 mL HCl + 0.8 mL HF

The precision was calculated on three replicates for all digestion procedures, with optimum digestion condition being 0.3 g. The sample was weighed in a Teflon vessel to which a mixture of 6 mL of HNO_3_, 3 mL of HCl, and 0.8 mL of HF was added, and the microwave oven was programmed 5 min 100 °C, 10 min 105 °C, and 75 °C for cooling. The residue was dissolved in 5 mL of HNO_3_ and, when necessary, the mixture was heated slowly to dissolve the residue and filtered for ICP-MS analysis. The solution was transferred to a 10 mL volumetric flask and made up to volume.

### 2.4 Method Validation

The method was validated by using A brand red tattoo inks. For the validation of the method, the accuracy, precision, limit of detection (LOD), limit of quantification (LOQ), linearity, range, sensitivity, and reproducibility were investigated.

The linearity of the method was determined by measuring five different standard solution mixtures. Standard solutions were prepared from 1000 μgg^–1^ stock solution of each element by dilution in different ranges. Each solution was injected three times.

The LOD is the lowest concentration of the analyte in a sample that can be determined by an analytical method. The calculations were done by analyzing blank solution. Measurements were made with the measurement of blank samples. LODs and LOQs were experimentally calculated as 3.3σ/*S* and 10σ/*S*, respectively, where σ is the standard deviation of the response of 10 blanks and *S* is the slope of the calibration curve (EURACHEM 2000).

Sensitivity is the capacity of the test process to record small changes in concentration. It is the slope of the calibration curve. The regression line determined in the linearity section is sufficient to determine the sensitivity.

Range is the limit of an analytical method where the process between the lowest and highest values is determined using the accuracy, linearity, and precision of a method by using standard solutions of each element.

Accuracy is defined as the proximity of the results to the real value. Accuracy of the method was determined with LGC7162-CRM (LGC, Germany). The systematic error was calculated, and then the *t*-test was performed to check the difference.

The precision of the method is the ability of the method to repeat any given value, or a degree of proximity of individual tests. Precision was evaluated in terms of intra and inter-run data distribution. The intra-run precision represents the same day repeatability of the data, and inter-run represents different day repeatability. The precision of the method was evaluated on standards, certified reference materials (CRMs), and real samples, and the results were given in RSD.

The blank level prior to measurement and instrumental drift were checked during the validation. Temperature differences and some of the unexpected anomalies could cause drift. Standard solutions were analyzed after every 20 samples and at the end of the analytical procedure. The drift percentage was calculated by the formula: *d*% = *C_n_* – *C_i_C_i_* × 100 where *C_i_* is the concentration (μgL^–1^) measured in the standard solution immediately after the calibration curve and *C_n_* is the actual concentration measured during the analytical sequence. A maximum *d*% of ±10% was considered acceptable for all elements.^10^

### 2.5 Chemometric Analysis

The JMP16 statistical software program was used for the chemometric analysis. The analytical results of the study were standardized in Excel 2016 and then imported to the JMP data table. ‘Data projection is performed mainly by methods principal component analysis (PCA),’ Otto,^11^ PCA was performed to the data, and the Score plot and Loading plot graphics were obtained for the chosen number of components. The hierarchical cluster analysis was performed for grouping the tattoos. The distances between the clusters are shown as a dendrogram. Dendrograms for cluster analysis are based on the Ward method.

## 3 Results and Discussion

Elements can be basically divided into two groups as essential elements for human health and nonessential metals classified as harmful to health. In this study, basic elements such as Al, Fe, Cu, and Zn were mainly evaluated. Ni and Co are important metals for the safe use of inks as they cause allergic reactions.^12^ Although they are essential for the body, excessive amounts of these elements can build up in the body and cause harmful effects. These metals can be detected in lymph nodes close to the tattooed areas. Inks applied under the skin can migrate through the body by blood flow. Various diseases, deformations, organ failures, and adverse effects have been reported in humans due to metal toxicity.^13−15^

During the tattooing process, a needle enters the skin with 50–3000 up and down movements per minute, allowing the ink to reach the dermis. The amount of ink applied to the dermis is approximately 1 mg of ink per cm^2^ of tattoo application area.^16^ Engel et al. calculated the mean amount of pigment in tattooed skin to be 2.53 mg cm^–2^ in an in vivo study.^17^ High absorption into the systemic circulation is expected for soluble compounds. However, the insoluble pigments used in tattoo inks remain predominantly in the dermis and then in the lymph nodes. The pigment particles remaining in the dermis cells form the colored skin. Tattoo inks applied under the skin cannot be considered as a cosmetic product. On the other hand, there is lifetime exposure. Risk assessments are different from cosmetic products because metals entering our body with tattoo applications are applied under the skin. The mixture of tattoo inks can transform into decomposition products and cause other chemical reactions. These unknown decomposition products and other transformation substances could be toxicologically active as well. As a result, since the different composition, chemical structures, and destiny of the inks in the body are not known exactly, it is difficult to evaluate their effects on health.^18^

### 3.1 Digestion

In our study, indicated elements (Al, Co, Cu, Fe, Ni, and Zn) have been detected in tattoo inks. At first, the influence of acid digestion treatment on elemental analysis was determined. For this purpose, six different mixtures of the acids were used as shown in Table 2. The mixture was selected based on references to strong acids used in tattoo inks, such as the study by Manso et al., 0.25 g of tattoo ink digested with 4 mL of nitric acid, 1 mL of hydrofluoric acid and 1 mL of hydrogen peroxide, and 0.2 g of ink digested with nitric acid (65%, w/w) and hydrogen peroxide (30%, w/w).^19^

As shown in Table 3, solvent mixture IV was found to be more appropriate for the sample preparation stage of real samples. A single acid solution was also used for digestion, but results were undetectable.

**Table 3:** Results of Using Different Solvent Mixtures for the Microwave Digestion (mg kg^–1^)

	Al	Co	Cu	Fe	Ni	Zn
I	2460 ± 31	0.16 ± 0.01	3.95 ± 0.22	133.22 ± 2.55	1.64 ± 0.05	39.58 ± 1.51
II	1528 ± 62	0.08 ± 0.01	1.90 ± 0.05	57.41 ± 2.75	2.46 ± 0.03	22.10 ± 0.93
III	1911 ± 47	0.40 ± 0.01	7.99 ± 0.31	72.66 ± 1.37	1.32 ± 0.05	11.41 ± 0.33
IV	1685 ± 26	0.03 ± 0.00	1.20 ± 0.33	61.58 ± 0.76	1.12 ± 0.02	9.29 ± 0.08
V	1648 ± 27	0.03 ± 0.00	1.36 ± 0.11	65.80 ± 0.76	0.87 ± 0.06	13.37 ± 1.23
VI	3437 ± 28	0.14 ± 0.04	3.91 ± 0.10	318.42 ± 2.77	0.89 ± 0.03	40.42 ± 1.53

### 3.2 Method Validation

Validation studies are based on the ICH guideline (EMEA, Note for Guidance on Validation of Analytical Procedures: Text and Methodology, CPMP/ICH/381/95, 1995, 1–15) within the scope of this study; Al, Co, Cu, Fe, Ni, and Zn were analyzed in tattoo ink samples.

The lowest correlation coefficient of all elements was 0.997. Standards were given to ICP-MS in the form of multielement analysis in accordance with its optimum range. Linearity, range, sensitivity, LOD, and LOQ of the analysis are given in Table 4.

**Table 4:** Some Validation Parameters of the ICP-MS Method for the Determination in Tattoos

elements	calibration equation	range (mg kg^–1^)	*R*	LOD (mg kg^–1^)	LOQ (mg kg^–1^)
^27^Al	*y* = 0.0017 × *x* + 0.0019	0–100	0.9998	28.15	85.305
^59^Co	*y* = 0.3045 × *x* + 0.0066	0–100	1.0000	0.0007	0.0020
^63^Cu	*y* = 0.2608 × *x* + 0.0397	0–100	1.0000	0.0043	0.0137
^56^Fe	*y* = 118.4017 × *x* + 4.1793	0–1000	1.0000	1.7674	5.3557
^60^Ni	*y* = 0.0966 × *x* + 0.0129	0–100	0.9999	0.0220	0.1337
^66^Zn	*y* = 0.0275 × *x* + 0.0894	0–100	0.9999	0.1433	0.4343

In our study, the precision parameter was obtained by standard solutions and Cr tattoo inks. It is stated in the International Council for Harmonization (ICH) guideline that internal data are obtained by reading each of the three concentrations covering a specific area three times. The results are given in Table 5. It has been observed that the precision of the method changes according to the element and the solution. Interday precision was lower than intraday precision, as expected.

**Table 5:** Precision and Accuracy Values of the Standard Solution, CRM, and Sample of Cr^a^

element	intraday run RSD %			interday run RSD %		accuracy
	100 (mg kg^–1^) standard	CRM	*C*(*r*)	100 (mg kg^–1^) standard	*C* (*r*)	*t*-test value *p* = 0.05
Al	0.6		0.8	4.8	6.8	
Co	4.4	1.4	2.2	6.2	14.3	1.4
Cu	3.9		4.1	8.2	8.1	
Ni	1.1	1.2	1.0	2.3	8.6	2.4
Zn	4.0	5.3	1.5	3.8	8.5	2.3

a Fe values could not be given due to the absence of Fe in the CRM.

Accuracy of the method can be checked for Co, Ni, and Zn. Systematic error was calculated and then the *t*-test was applied. There is no significant difference between the CRM and sample (*p* ≤ 0.05).

Microwave-digested tattoo ink samples were analyzed in ICP-MS using an internal standard after appropriate dilutions. The results are given in Table 6.

**Table 6:** Content of Metals in Tattoo Inks Analyzed in This Study^a^

color	brand	Al	Co	Cu	Fe	Ni	Zn
green	A	2583 ± 15	0.105 ± 0.004	1672 ± 33	87.33 ± 1.00	3.10 ± 0.02	9.68 ± 0.77
	B	1979 ± 13	0.088 ± 0.005	2523 ± 90	66.72 ± 0.60	1.00 ± 0.02	22.74 ± 0.77
	C	1984 ± 33	0.043 ± 0.006	213.6 ± 3.1	16.98 ± 0.04	0.63 ± 0.04	21.33 ± 0.50
black	A	1933 ± 16	0.223 ± 0.003	4.38 ± 0.17	254.17 ± 2.63	2.83 ± 0.02	36.58 ± 1.28
	B	1843 ± 15	0.300 ± 0.026	1.98 ± 0.09	126.02 ± 0.43	4.21 ± 0.01	15.92 ± 0.78
	C	2171 ± 45	0.331 ± 0.023	1.24 ± 0.08	145.15 ± 3.56	3.98 ± 0.09	14.33 ± 0.92
red	A	3425 ± 28	1.065 ± 0.024	3.77 ± 0.11	318.42 ± 2.77	17.53 ± 0.10	46.90 ± 0.72
	B	1191.1 ± 9.8	0.044 ± 0.002	72.27 ± 4.80	62.75 ± 0.81	0.93 ± 0.02	2.62 ± 0.22
	C	1245 ± 37	0.126 ± 0.006	1.49 ± 0.05	125.15 ± 1.56	3.87 ± 0.04	28.05 ± 0.88

a Values are expressed as mg kg^–1^ (mean ± standard deviation of three replicates).

There are limited studies regarding the element content of tattoos, especially in Turkey. Piccinini et al. published the JRC report5 and reported Co, Cu, and Zn values as 6.8, 31.8, and 21.6%, respectively in tattoo inks.^20^ The comparison of the references about elemental analysis in tattoo inks is given in Table 7. In this study, the amount of elements in tattoo inks showed great variations among some colors and some brands. This is particularly notable for the element Cu.

**Table 7:** Trace Element Levels in Tattoo Inks: Reference Comparison^b^

		Al	Co	Cu	Fe	Ni	Zn
Eghbali et al.^49^	green				17.74		5.23
	black				9.29		5.43
	red				15.79		1.23
Battistini et al.^31^	green	11.4		0.22	20.8		0.098
	black	7.93		n.d.	11.4		0.42
	red	9.23		n.d.	6.35		2.65
Forte et al.^4^	green	254	0.110	5887	44.9	5.049	
	black	1.92	0.072	10.4	5.47	0.073	
	red						
	green^a^	2012	0.024	1606	19.6	0.258	
	black^a^	9.36	0.011	5.02	6.42	0.070	
	red^a^	670	0.009	1.47	38.5	0.067	
	green	1960	0.025	45.4	54.5	0.154	
	black	189	0.013	0.79	69.3	0.087	
	red	2.41	0.011	0.79	0.72	0.045	
Forte et al.^43^	green		0.096			2.318	
	black		0.025			0.424	
	red		0.017			0.179	
Manso et al.^19^	green			4400		n.d.	
	black			6		3	
	red			61		n.d.	
Arl et al.^45^	green	1323.54		724.45	17.74		
	black	13.16		0.71	9.29		
	red	13.07		0.35	15.79		
this study	green^a^	1978.9	0.088	2523.4	66.7	1.0	22.74
	black^a^	1842.9	0.300	1.98	126.0	4.2	15.92
	red^a^	1191.1	0.044	72.27	62.75	0.9	2.62
limit of ResAP (2008)1				25	25		50

a The same brand.

b The results are given in mg kg^–1^.

The amount of Cu in green in inks was higher in all three brands. The reason why the amount of Cu in green inks is higher than that of red and black ink tattoos is thought to be related to the color factor and the amount of elements it contains in tattoo inks. While the amount of Cu is higher in green and red colored tattoo inks in B brand, this amount was higher in A brand in black colored inks. Cu is a metal with the capacity to initiate oxidative damage in cells and is thought to induce cellular toxicity.^21^ The limit value for Cu was determined as 25 mg kg^–1^. It is seen that this limit is exceeded in the analyzed samples, especially in green inks (213.6–2523.4 mg kg^–1^).

Cu is an essential mineral. Data on dermal toxicity caused by Cu compounds are insufficient. It is reported in EC regulation 1272/2008 that CuSO4 is skin irritant 2 ((ResAP 2008)1).^9^

Li et al., in their study, determined the irritating effect of Cu on the skin. However, copper-peptide (GHK-Cu) has low potential to cause skin irritation and therefore offers a safer alternative to the transdermal delivery of copper.^22^ There are also some papers related to the contact dermatitis effect of Cu.^23,24^

Al concentration was determined in the range of 1191.1–3424.9 mg kg^–1^ in all analyzed samples, and it is quite high compared to all other element amounts. Al content was found to be close to each other in all color inks of B and C brands. Al has been found in components of some inks as cobalt aluminate. Al salts are used in red and purple inks. In a study of 30 tattoo inks, 87% reported the presence of Al.^25^ Co and Al are known to cause granulomatous reactions^26,27^ It is reported in EC Regulation 1272/2008 that AlCl_3_ is skin corrosive 1B ((ResAP 2008)1).^9^ Exposure of the skin to low doses of aluminum chloride for 18 weeks has been shown to result in aluminum accumulation in the brain.^28^ Moreover, it has been observed that intradermal injection of aluminum salts increases granuloma formation. Zn has an irritating effect on the skin as well.^28,29^ Al skin penetration is insignificant in healthy individuals, but important in shaved adults. While the injected Al dose is desired to be 25 g L^–1^, the maximum parenteral dose not to exceed 1 mg kg^–1^ day so that Al does not accumulate in the blood circulation.^30^

Zn is an essential element for many intracellular molecular reactions and may play an important role in the induction of apoptosis. It is hypothesized that apoptosis induces the toxicity of cadmium through its interaction with the Zn finger protein. When the Zn contents of different brands and colors were evaluated, it was determined that Ar ink had the highest Zn content (46.90 mg kg^–1^). Today, ZnO is used as a UV filter in sunscreens, as well as in creams to relieve skin damage such as burns, wounds, and irritations. It is reported that the use of this compound is safe. Zinc chloride is listed as an inactive ingredient in FDA-approved drug products to be administered subcutaneously (0.006%) and intradermally (0.7%).^31^ In EC Regulation 1272/2008 ZnCl_2_ is reported to be corrosive to skin ((ResAP 2008)1).^9^

In tattoo inks, Fe forms red (Fe_2_O_3_), black (Fe_3_O_4_), yellow (FeOOH), and brown (iron oxide mixture) colors in different formulas. Iron oxide is a known darkener used in tattoo inks. These iron oxides are present in inorganic inks, albeit in small quantities. It is reported that it reacts with O_2_ and H_2_O and turns into different salts. Iron oxide formation has been associated with significant deleterious effects, such as inflammation, apoptosis, disruption of mitochondrial function, membrane changes, reactive oxygen species formation, increased micronucleus induction, and chromosome condensation, depending on concentration, exposure time, and cell type.^32^

It was emphasized that iron accumulation due to iron oxide compounds caused a decrease in the GSH level in neural tissues and induction of oxidative stress. In addition, it has been reported that iron oxide-based pigments can react during magnetic resonance imaging scans and trigger low-grade burns^33^ Dixon et al. reported that iron accumulation causes an increase in cytotoxic lipid oxidation in the cell.^34^ Likewise Imam et al. reported that iron oxide nanoparticles cause damage to the membrane of rat brain endothelial cells by producing ROS.^35^

It is also reported that iron oxide pigments always contain a small amount of Ni as an impurity. It is interpreted that Ni may cause allergic reactions. Fe concentration was determined in the range of 16.98–318.42 mg kg^–1^ in all analyzed samples in this study. The highest Ni concentration was found in the Ar sample as 17.53 mg kg^–1^. ResAP(2003)2 and ResAP(2008)1 define the limit value for Ni as ‘as low as technically achievable’.^8,9^ Ni is an immunotoxic, neurotoxic, and carcinogenic agent. Depending on the dose and exposure time, it can cause a variety of effects, including contact dermatitis, cardiovascular diseases, asthma, lung fibrosis, and respiratory tract cancer. Ni causes oxidative stress and mitochondrial dysfunctions. Chronic exposure causes accumulation of nickel and nickel compounds in the body. Nickel^2+^ exposure has been associated with DNA hypermethylation and transcriptional repression of tumor suppressor genes in vitro and in vivo. It has been reported that nickel ions trigger apoptosis by acting on caspases in the cell. Intradermal nickel exposure can cause scaly red areas and localized erythematous, pruritic vesicles.^36,37^

Cobalt is an essential element required for vitamin B12 synthesis in the body. However, high levels cause adverse effects. Cobalt has no place in the diet. It is taken 5–40 μg daily with nutrition. Its total level in the body is estimated to be between 1.1 and 1.5 mg.^38^ It causes adverse effects when taken in high doses. Cobalt can cause allergic contact dermatitis, eye irritation, and prolonged contact sensitization.^39^ The International Agency for Research on Cancer has listed cobalt and cobalt compounds as agents that are possibly carcinogenic to humans (Group 2B). A study in rabbits exposed to cobalt chloride at a dose of 1354 μg/mL for 18 days reported marked neuropathy with demyelination and atrophy of the optic nerves.^38^ It has been reported that cobalt ions cause oxidative stress and cytotoxicity by generating a large number of reactive oxygen species and HO radicals in cells.^40^ Cobalt ions and cobalt nanoparticles were found to be effective on cell signals, enzymes, and cell metabolism. It has also been shown to interact with various receptors, ion channels, and biomolecules in cells.^40,41^ It has been reported that cobalt ions can replace essential metal ions by interacting with metal-based proteins in the cell (e.g., Mg^2+^, Ca^2+^, Zn^2+^, etc.), thus causing dysfunction in these enzymes or proteins.^42^ All these findings support the idea that cobalt ions and nanoparticles can cause cell death due to oxidative stress. There is no FDA-specified limit value for cobalt in tattoo inks (ResAP(2003)2 and ResAP(2008)1). Co concentration was determined in the range of 0.04–1.07 mg kg^–1^ in all analyzed samples in this study.

In 2009, Forte et al. conducted a survey with tattoo inks of different brands and colors.^4^ One of the brands that were used in this study is the same brand that is used in our study. When the data from the two studies were compared, it was seen that the amounts we obtained in general were higher.^43^

Lim and Shin analyzed Al, Co, Cu, Fe, Ni, and Zn in tattoo inks without classifying the color. The results were 0.1, 1.7, 1840, 24,700, 5, and 8.7 mg kg^–1^, respectively. Cu, Fe, and Ni values were higher than those in this study.^44^

In the study by Arl et al., high concentrations of Al and Cu levels were identified in green inks in tattoo inks, although not specified on the labels. Cu and Fe are commonly applied in small amounts in black and red inks.^45^

In Turkey, there are few studies on this topic. Kılıç et al. worked toxic metals in some cosmetic products consumed in Turkey and they found a concentration of Co 0.1 and 0.9, Cu 0.3 and 2.2, and Ni 0.3, 2.5, and 3.3 mg kg^–1^.^46^ In another study, Co, Cu, and Ni were determined in finger paint samples in Turkey, and, for red color paint Ni concentration was found to be 1.5 mg kg^–1^ and in green paint Cu and Ni concentrations were found to be 0.5 and 2.3 mg kg^–1^, respectively. The other elements could not be detected in this study.^47^ The amounts of the elements vary according to the type and color of the material.

Some studies were done in biopsies from tattooed skin samples to find the effect of some elements coming from tattoos on skin. Serup et al. analyzed Cu, Fe, and Ni in both tattoo inks and biopsies.^7^ They found 1608.7, 2317.8, and 0.7 and 42.93, 3.48, and 1.05 mg kg^–1^, respectively. They had higher results with tattoo inks overall. De Cuyper et al. found 4.3, 5.9, 21, and 0.4 mg kg^–1^ of Al, Cu, Fe, and Ni, respectively, in biopsies taken from tattooed skin. As can be seen, the results in biopsy samples are different from each other.^48^ Considering all these evaluations, it can be said that the amount of trace element varies depending on the color and brand.

### 3.3 Chemometric Analysis

**Figure 1:**
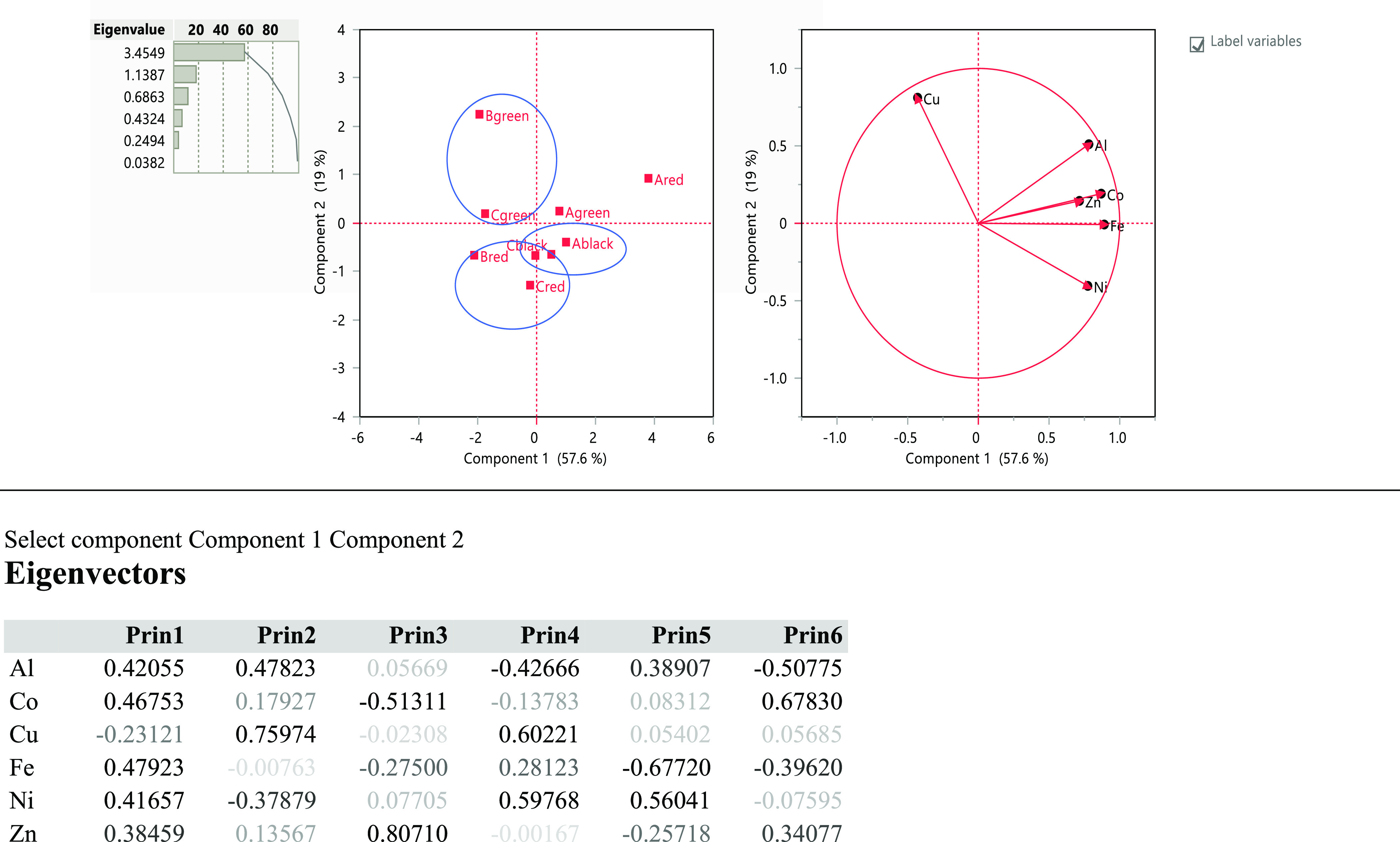
PC analysis of some elements in tattoo inks. Eigenvectors, eigenvalues score, and loading plots.

Multivariate analysis was performed for the classification of the tattoo inks according to their elemental pattern. The tattoo data in Figure 1 show that 57.6% of data variance can be explained by use of two principal components (Figure 1). PCA on the basis of correlation matrix of the data provides the results given in Figure 1 for the scores and loadings. It has been possible to group the different brand of tattoos according to their color by the score plot. The loading plot provides the projection of the features on the principal components. There is the greatest correlation for the elements Zn and Co, both on the same line. The features have correlation except Cu.

**Figure 2:**
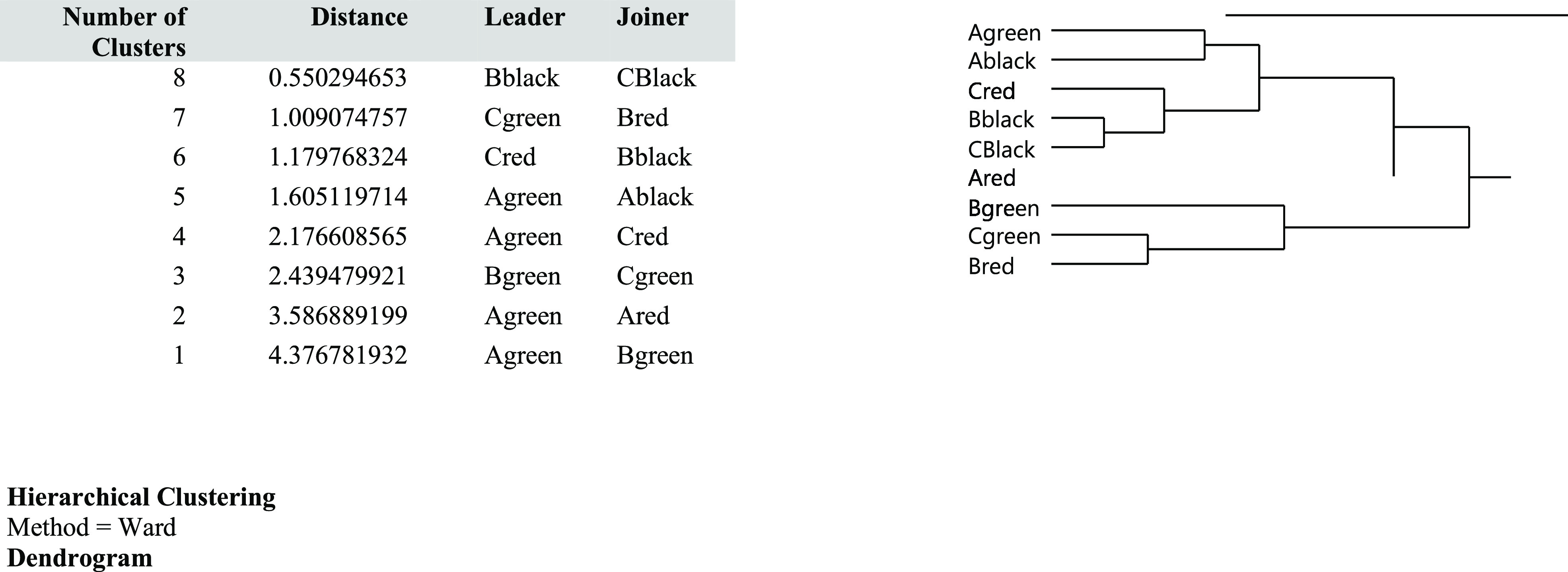
Cluster analysis of some tattoo inks according to the elemental structure: distance and dendrogram.

The dendrogram obtained by cluster analysis using the Ward method for raw ICP-MS data (Figure 2) distinguished the data. It can be said there are two main groups in the data. Ag, Ab, Cr, Bb, Cb, and Ar are in one group and Bg, Cg, and Br in the second group. Bb and Cb have highest correlation and Ar is the worst.

## 4 Conclusions

Validated trace element determination is very important in health care analysis. In this study, amounts of some metals were measured in samples taken from the market, and some of the amounts were found to be above the concentrations specified in the guidelines and in amounts that could pose a risk to public health. Reliable results were obtained by validating the method and choosing the best acid combination for the preparation of the samples.

Many people may not be aware that they can get harmful effects by the chemicals of tattoo inks. We believe that it will be beneficial to make the results open to public and to inform tattoo artists and people who have tattooed, albeit limited, when deciding to purchase tattoo ink.

A large number of people today have one or more tattoos. While manufacturers need to comply more with tattoo safety laws, potential tattoo risks need to be taken more seriously. Consumer protection measures are needed in every country.

In the risk assessment of tattoo inks, evaluations should be made with realistic scenarios and safe substances, and their amounts should be determined up to a defined dose for intradermal application.

Besides regulation, standardization is also an important element in the use of tattoo inks and high-purity chemicals for tattooing. Normalizing standardization at the national and international level can help in terms of quality and public safety. Finally, awareness of tattoo artists and people who get tattoos should be raised about the lifetime exposure to these ingredient mixes for potential adverse health effects as well as being an aspect of art.

## Abbreviations

Co: cobalt
Al: aluminum
Cu: copper
Zn: zinc
Fe: iron
Ni: nickel
CoE: ResAP The Council of Europe Resolution
ICP-MS: inductively coupled plasma-mass spectrometer
LOD: limit of detection
LOQ: limit of quantification
JRC: Joint Research Centre
ICH: International Council for Harmonization
CRMs: certified reference materials
